# Why have so many African leaders died of COVID-19?

**DOI:** 10.1136/bmjgh-2021-005587

**Published:** 2021-05-17

**Authors:** Jean-Benoît Falisse, Robert Macdonald, Thomas Molony, Paul Nugent

**Affiliations:** 1 Centre of African Studies, The University of Edinburgh, Edinburgh, UK; 2 Edinburgh Futures Institute, The University of Edinburgh, Edinburgh, UK; 3 School of History Classics and Archaeology, The University of Edinburgh, Edinburgh, UK

**Keywords:** COVID-19, health policy, descriptive study

## Abstract

This paper provides evidence that the COVID-19-related mortality rate of national government ministers and heads of state has been substantially higher than that of people with a similar sex and age profile in the general population, a trend that is driven by African cases (17 out of 24 reported deaths worldwide, as of 6 February 2021). Ministers’ work frequently puts them in close contact with diverse groups, and therefore at higher risk of contracting SARS-CoV-2, but this is not specific to Africa. This paper discusses five non-mutually exclusive hypotheses for the Africa-specific trend, involving comorbidity, poorly resourced healthcare and possible restrictions in accessing out-of-country health facilities, the underreporting of cases, and, later, the disproportionate impact of the so-called ‘South African’ variant (501Y.V2). The paper then turns its attention to the public health and political implications of the trend. While governments have measures in place to cope with the sudden loss of top officials, the COVID-19-related deaths have been associated with substantial changes in public health policy in cases where the response to the pandemic had initially been contested or minimal. Ministerial deaths may also result in a reconfiguration of political leadership, but we do not expect a wave of younger and more gender representative replacements. Rather, we speculate that a disconnect may emerge between the top leadership and the public, with junior ministers filling the void and in so doing putting themselves more at risk of infection. Opposition politicians may also be at significant risk of contracting SARS-CoV-2.

**Summary box**

- In the 12 months between 6 February 2020 and 6 February 2021, COVID-19 claimed the lives of at least 24 national ministers and heads of state, which is well in excess of the reported in-office death rate for such politicians in recent years. Seventeen of these deaths are from the African continent (and the count kept growing after 6 February 2021), putting the COVID-19 death rate at 1.33% among national ministers and heads of states—seven times above estimates of the world's average for a demographic profile of similar sex and age average for the same period.
- While comorbidities such as obesity or diabetes cannot be ruled out as a cause of such African specificity, other hypotheses include the lower quality of healthcare and a halt in international medical transfers used by African elites as well as, later, the incidence of the so-called ‘South African’ variant (501Y.V2) of the virus.
- The trend also invites careful reflection on the spread of COVID-19 in countries where data and testing are limited, especially among similar demographic profiles.
- The deaths have an important symbolic value and have been associated with shifts in COVID-19 policies in some countries. They are also likely to result in some reconfiguration of the political space.

## INTRODUCTION

As the COVID-19 pandemic enters its second year, there is an ample and growing body of evidence that certain demographic factors, such as age and biological sex, affect a person’s susceptibility to the SARS-CoV-2 virus.^1 2^ Socioeconomic factors, including people’s occupations, also impact the likelihood that someone is exposed to SARS-CoV-2, particularly when jobs are not adaptable to working from home and involve close contact with other people.^3^ Although these jobs are most likely to be held by low-income people, this description arguably applies to political elites. There have been several high-profile cases of politicians becoming ill with COVID-19. However, accurately determining political elites’ susceptibility to COVID-19 requires systematic investigation.

In this paper, we present evidence that the COVID-19-related mortality rate in national governments is substantially higher than that of the general population, mostly owing to deaths in African countries. We show that sex and age do not fully explain this trend, before exploring possible explanations and discussing public health and political consequences.

## How can we know whether politicians are more likely to die of COVID-19?

The health and well-being of those in positions of power are typically the subject of much media attention. In the analysis below, we focus on ministers and heads of state. They represent only a fraction of those in positions of power, but they also constitute a well-defined group that can be counted in a similar fashion across countries. We created a list of deaths based on two sources that were each systematically verified. First, we looked at lists appearing in traditional online media—especially Deutsche Welle (Germany),^4^ The Citizen (Tanzania)^5^ and Al Jazeera (Qatar)^6^—that mention prominent COVID-19-related deaths. Second, we sorted through Google News results generated by the keywords ‘minister’, ‘died/dies’ and ‘COVID-19/coronavirus’, in English and French. The high visibility of ministers and heads of state means that the list is likely to be comprehensive, but the suppression and politicisation of information on prominent COVID-19 cases remains an issue. Presidents Magufuli of Tanzania and Nkurunziza of Burundi are discussed in the paper because, while the communiqués announcing their deaths do not mention COVID-19, many in the media and civil society have suggested such a (plausible) connection.

For this reason, and because it is less likely to make the news, listing people who tested positive for COVID-19 is more challenging. We focused on Africa and compiled a list using data from Jeune Afrique magazine,^7^ as well as results from manually sorting through Google News and systematically checking the articles containing ‘minister tested positive’ and ‘COVID-19’ on Factiva.

In January 2020, the Inter-Parliamentary Union and UN (IPU/UN) Women released a count of women in ministerial positions in 190 countries.^8^ It shows 78% of ministers are men and provides the number of people holding ministerial portfolios in each country. In addition to sex (sex corresponds to gender for most ministers), age is a key factor determining susceptibility to COVID-19: the WhoGov dataset, which includes information on ministers in 177 countries for the period 1966–2016, was the best available source.^9^ Our focus is on national-level (or federal/union) ministers and heads of states. Data on COVID-19 mortality disaggregated by sex, age and country are available for 54 countries from the Sex, Gender and COVID-19 Project.^10^

## African ministers are more likely to die of COVID-19

We identified 24 deaths that occurred between 6 February 2020 (the first reported COVID-19 death outside Asia) and 6 February 2021 (see online supplemental file). We focus the statistical analysis on these 12 months.

10.1136/bmjgh-2021-005587.supp1 Supplementary data

**Figure 1:**
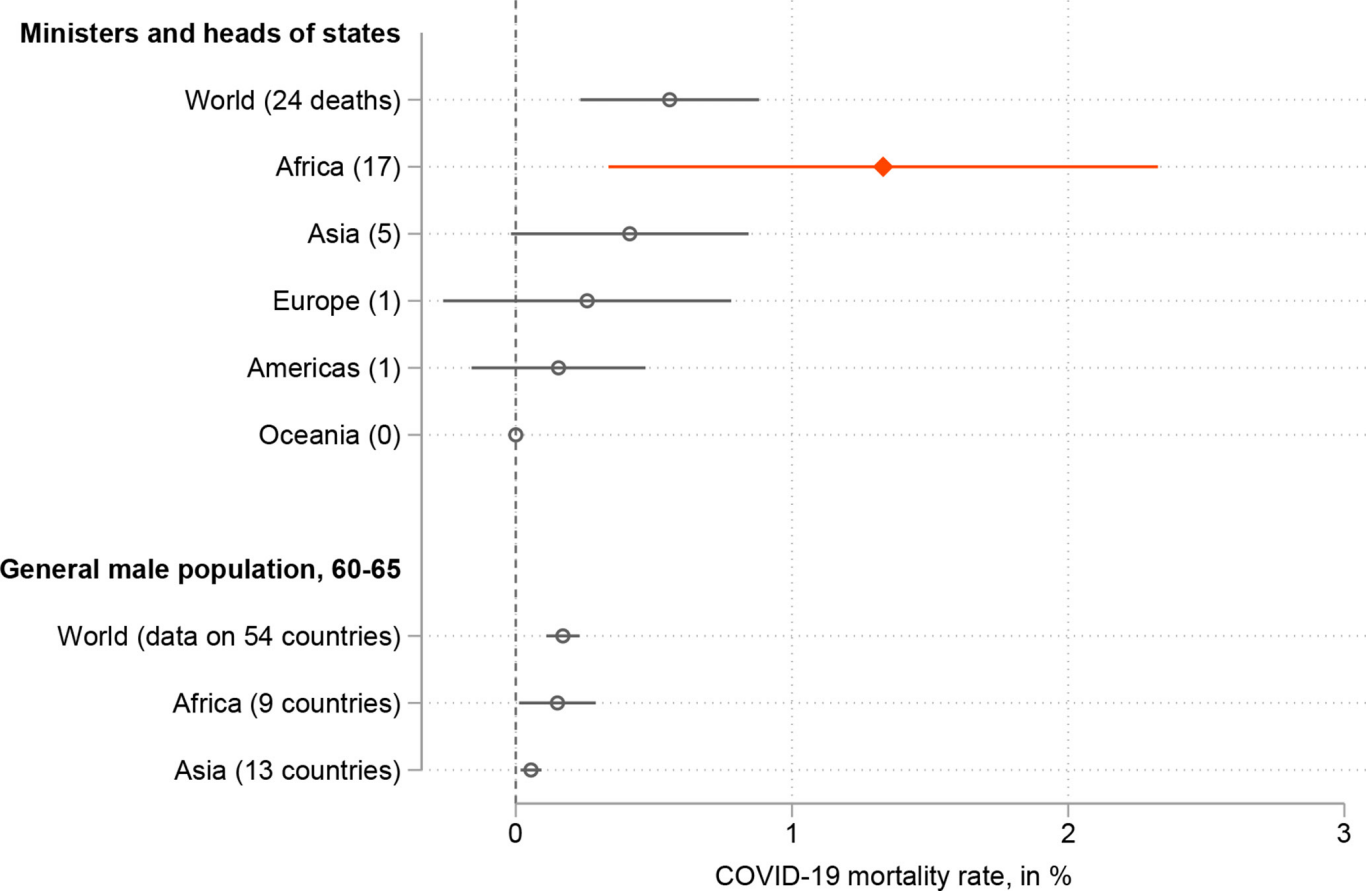
COVID-19 death rates (as of 15 February 2021). Data sources: ministers and heads of states—deaths: authors’ own compilation/total number of ministers: IPU/UN Women (01 January 2020). General death rate—Sex, Gender and COVID-19 Project (15 January 2021), see text for details. Bars are 95% CI.

Using the IPU/UN Women data, this suggests a mortality rate of 0.60% among ministers and heads of state (figure 1). It is significantly different from the general mortality rate for a population similar to the ministers’, that is, 60.5 years old and men, which our highest estimate puts at 0.17% (using the 56 countries for which we have data on both groups: t(53)=1.780, p=0.087). In 48 countries out of 56, 60.5 years is included in a bracket that goes up to at least 70 years old and Europe is disproportionately represented (23 countries).

The WhoGov dataset contains the year of death for some ministers and heads of state. In the most recent period (2010–2016), which covers only 101 countries, an average of 0.24% (SD: 4.894) of them died during a year when they were in office (which does not necessarily mean they died in office). We do not have a count of ministers and heads of state who died of causes unrelated to COVID-19 for 2020 or 2021. However, when counting only COVID-19-related deaths, the excess mortality would already be around 140% of our estimate for 2010–2016.

The death toll is particularly high in Africa. The 17 deaths give a mortality among ministers and heads of state of 1.33%, which is substantially higher than both the global death rate for people of similar age and gender (t(105)=2.357, p=0.023) and the African figure that stands even lower at 0.15%. The latter statistic comes from only nine countries, and its accuracy is likely limited (especially outside urban areas. The ministerial death rate in these nine countries is incidentally substantially higher, at 2.5%). The results are similar when using the 12-month period before 30 March 2021 (submission of the revised version of this manuscript). Overall, Zimbabwe (four deaths) and eSwatini (three deaths, including the prime minister) are the most affected.

According to the partial data from the WhoGov dataset, the average age of African ministers was 63.93 (SD 6.25) in 2016. This is slightly higher than that of the rest of the world combined at 60.09 years old, but is not statistically different from Asia’s (62.93) or the Americas’ (62.41). The level of gender parity among African ministers is similar to the global level (23% women vs 21% for the rest of the world combined). Those African ministers and heads of state and government who died of COVID-19 were all men, with the exception of Ellen Gwaradzimba (Zimbabwe’s minister of State for Manicaland Provincial Affairs), and on average they were aged 61.22 (SD: 9.48). If anything, the African leaders who succumbed to COVID-19 were slightly younger than their seven counterparts on other continents (62.66; SD: 7.71).

Providing an accurate estimate of the case fatality rate among ministers and leaders is difficult because of the likelihood our list of positive cases is incomplete. Focusing on the most preoccupying case of Africa, the available data give a rather unrealistic 26%. Using the infection fatality rate may be more informative: the 1.33% mortality rate reported above is similar to the global infection fatality rate for men aged 65 estimated by O’Driscoll *et al* based on 45, mostly high-income, countries.^11^

## Why do so many African leaders die of COVID-19?

High-level politicians are likely to be exposed to SARS-CoV-2 more often than the average citizen in their countries. They are typically in close physical contact with a lot of people, as their jobs involve formal meetings, parliamentary sessions and interactions with the media and the general public. They also tend to travel a lot, both domestically and, when possible, internationally. They meet many people from diverse groups, some of whom, including other politicians, are also in high-risk categories—COVID-19 clusters have been found among the cabinets of Guinea Bissau in March (five positive cases, including the prime minister),^12^ South Sudan in May 2020 (10 positives)^13^ and Zimbabwe in January 2021 (three ministers died).^14^ Many of the leaders’ activities are difficult to shift online, and often involve being indoors, where the likelihood of contracting SARS-CoV-2 is far higher. It is also worth noting that the first COVID-19-related ministerial deaths were in countries where the parliament was still meeting in-person in the first half of 2020, such as Somalia, Guinea, Niger and Burundi.^15^ Elections involve rallies and even more meetings, and therefore further interpersonal contact and risk of infection: in Africa, over 40% of the countries that had an election in the last 12 months lost at least one minister to COVID-19, versus only 10% for countries that did not (χ^2^(1, n=53)=7.154, p=0.007). For example, in Guinea, which held a referendum in March 2020 and a general election in October 2020, Minister Secretary General of the Government Sékou Kourouma died of COVID-19 in April, only 24 hours after the head of Guinea’s electoral commission also succumbed to the disease.^16^

Given that these occupational risks exist in all countries, they cannot account for why the number of senior political figures who have died on the African continent during the pandemic is relatively high. As their sex and age profiles also do not fully account for this, further explanation is required. We will now investigate five non-mutually exclusive hypotheses.

A first possibility is that African leaders are more likely to exhibit COVID-19 comorbidities. The evidence to support this hypothesis is limited and is generally not reflected in publicly available information: individual comorbidities are typically kept confidential and, even after death, they are rarely made public. Furthermore, a study based on high-income countries has established that people elected to their country’s highest office tend to die younger—a phenomenon described as ‘accelerated ageing’—but no specific health conditions are mentioned.^17^ However, we uncovered no evidence that indicates this issue affects African leaders more than their counterparts in other regions. Diabetes and obesity are more frequent among wealthy African individuals with a high level of education,^18^ but this is also the case in Asia.^19^ It was notable that at least five of the senior politicians who died after becoming ill with COVID-19 were known diabetics, including two Bangladeshi ministers and eSwatini prime minister Ambrose Dlamini.

A second hypothesis relates to healthcare provision. Many African health systems are chronically under-funded and under-resourced, and this hampers their response to COVID-19 infections.^20^ While US president Donald Trump benefitted from cutting-edge experimental treatment when he was in office, this may not have been the case for many African leaders. African elites, especially those from the poorer nations, often seek to access better healthcare in higher-income African countries or in private clinics in Asia or Europe.^21^ For example, former president Robert Mugabe regularly took his medical check-ups in Singapore, where he died in hospital in 2019. During the same year, current Zimbabwean vice president and minister of health Constantino Chiwenga spent 4 months receiving medical treatment in China.^22^ There is no research on whether medical transfer abroad was formally prohibited, but restrictions of movement in the context of COVID-19 may have prevented African elites from obtaining the better-quality healthcare that they would normally have accessed,^23^ especially at short notice. Prime minister Dlamini from eSwatini was transferred to South Africa, but not outside the continent. Algerian president Abdelmadjid Tebboune was admitted to a German hospital in November 2020 and survived.^24^ All the African ministers who died of COVID-19 died in their country of origin.

A third possibility is that mortality in the general African population is higher than reported. This argument is made in recent publications,^25^ but has been challenged by the WHO^26^ and studies that use several demographic, environmental and cultural features to explain the comparatively low death rate in Africa.^27 28^ As mentioned above, the ministers and heads of state considered in this analysis are particularly exposed, and it would be dangerous to infer too much about the broader population. They may, however, represent a wider class of politicians and public personalities, as there is substantial anecdotal evidence of other high-profile individuals dying of COVID-19 across Africa: examples include Mali’s leader of the opposition Soumaila Cissé,^29^ Zanzibar’s first vice president Maalim Seif Sharif Hamad,^30^ Nigerian president’s chief of staff Abba Kyari^31^ and Zimbabwe’s high court judge Clement Phiri.^32^ At a more general level, the extremely high case fatality rate we compiled using publicly available data does suggest under-reporting of positive cases highlighted in various publications, which may be associated with the relatively low levels of testing in many African countries.^33^

A fourth possibility is that the risk of contracting SARS-CoV-2 is higher for African ministers because their work environment is busier and, therefore, more prone to the circulation of the virus. African cabinets are on average larger than those in the rest of world (26.05 vs 19.13 ministers on average according to the IPU/UN Women dataset: t(188)=5.785, p=0.000. The difference holds when comparing to Asia, which comes second, and when using the WhoGov 2010–2016 data). However, it remains a weak hypothesis in the absence of comprehensive comparative data on ministerial cultures across continents—including, for instance, travel, meeting and rally habits and ministerial cabinet sizes.

Finally, it is worth pointing out that no fewer than 9 out of the 17 death cases in Africa are in Southern Africa (in South Africa, eSwatini, Zimbabwe and Malawi), and eight of them occurred after the so-called ‘South African’ SARS-CoV-2 variant (501Y.V2), which is more transmissible,^34^ was first reported.

## Consequences: shaping health policy… and democracy?

States have well-established procedures to deal with the deaths of ministers and heads of state while in office, and we did not find evidence of significant destabilisation of governments following these COVID-19-related deaths. However, the deaths do have an important symbolic impact on the population and government, and they have been associated with substantial changes in public health policy, especially in contexts where the response to the pandemic had been contentious or minimal. In Malawi, where the country’s high court had blocked lockdown measures in April 2020 following a challenge from civil society groups,^35^ the death of Lingson Belekanyama (minister of local government and rural development) was used by president Lazarus Chakwera to stress the importance of new restrictions.^36^ In Burundi, president Nkurunziza died on 9 June 2020 following a short stay in hospital. Despite the official communiqué mentioning ‘cardiac arrest’, it is strongly suspected that COVID-19 was the cause of death.^37^ Hitherto, the Burundian authorities had downplayed the severity of the epidemic in the country and the WHO representative had been expelled for allegedly interfering with pandemic management. By 30 June, new president Evariste Ndayishimiye had announced that COVID-19 was Burundi’s biggest enemy, and a new campaign of active COVID-19 screening was launched shortly after.^38^ In December 2020, when former president Pierre Buyoya died, there was no hesitation in announcing that it was due to COVID-19.^39^ Tanzania’s president John Magufuli made global headlines for minimising the danger of COVID-19 and calling for prayers and untested herbal medicine as treatment and prophylaxis.^40^ In May 2020, the country stopped reporting cases, and on 1 February 2021 the health minister announced that Tanzania was still not interested in a vaccine programme.^41^ However, on 19 February, the president’s chief secretary, John Kijazi, was among a series of prominent people who died of what many in the media suspected was COVID-19.^42^ On 22 February, president Magufuli finally urged citizens to wear masks.^43^ These events were soon overtaken by his own death, which, according to official sources, occurred on 17 March due to heart complications. Before it was announced, Magufuli had been absent from public view for several weeks. During this time, rumours that he had COVID-19, and that he had gone to Kenya for treatment, circulated widely.^40^ At the time of writing, it is early in new president Samia Suluhu Hassan’s tenure, but there are indications that further changes to COVID-19 policy will be implemented, including the recent publication of new ministry of health COVID-19 treatment guidelines. Countries that already had strong COVID-19 responses in place, such as Zimbabwe, Niger or Senegal, saw no substantial changes to health policies following ministerial deaths.

Ministerial deaths will result in a reconfiguration of political leadership. However, the passing of the baton to a younger or more gender representative cohort is unlikely (with President Samia in Tanzania being a notable exception). The number of fatalities has been too low to have such a catalytic effect and, as the case of Zimbabwe shows, there are many more politicians, with similar profiles, waiting to fill gaps.^41^

There may be a shift in the manner in which government business is transacted. The image of leaders who have prided themselves on their longevity and indestructability is likely to be tarnished as they die or disappear from public view—although none of the most long-lasting presidents has yet been a casualty of COVID-19. It is possible that as they become less visible in public, more of the work of government will fall to deputies and junior ministers who exist in significant numbers. The disconnect between the top leadership, the rest of the government apparatus and the wider public is likely to increase. Junior or deputy ministers may be somewhat younger, and, in many countries, these positions are occupied by women more frequently than senior positions. While there is a reduced chance of mortality, they are likely to be much more exposed than the substantive ministers who carry out fewer daily interactions even though they ultimately call the shots.

The death of a significant number of opposition politicians—who are also highly itinerant and interact with many people—could spell a reduction in the pool of those who are prepared to challenge and call to account the sitting government. The same can be said for lawyers (as seen in the case of the Tanganyika Law Society^42^), and other public figures, such as academics, who in many African countries play an important public role in ensuring that there are political checks and balances, and have already suffered major casualties in countries such as Zimbabwe,^43^ Tanzania^44^ and Nigeria.^45^

African lawmakers across the political spectrum are of course aware that many of their number are succumbing to the pandemic, and most African countries are calling for an accelerated rollout of foreign-made vaccines. Some political elites, it has been rumoured, have already had privileged access to vaccines,^34^ which, if true, suggests that they are not sceptical about immunisation—and this bodes well for political leaders acting as role models for the wider population when further vaccines become available. This is now the case in Ghana and Ivory Coast, where mass inoculation has begun with assistance from the UN-backed Covax distribution initiative. Mindful perhaps of their vulnerability, Ghana’s president Nana Akufo-Addo, and Ivory Coast’s minister of culture Raymonde Goudou Coffie, were at the front of the queue.^46^

## Data Availability

The IPU/UN Women, WhoGov, and Sex, Gender and COVID-19 Project data are available in public open access repositories. Our own compilation of COVID-19-related deaths and positive cases is included in the online supplementary information.
