# Supplementary material for: Why have so many African leaders died of COVID-19?
Source: BMJ Glob Health. 2021 May 17;6(5):e005587. doi: 10.1136/bmjgh-2021-005587 (PMC8130489; doi:10.1136/bmjgh-2021-005587)
Supplement: Supplementary data [file bmjgh-2021-005587supp001.pdf]

For full dataset (Stata) see DOI 10.17605/OSF.IO/KFJVQ

Ministers and heads of state who died of COVID-19 (6 February 2020 – 6 February 2021)

date of death	***country***	***name***	***age***	***occupation***	***place of death***	***source***
**12-Apr-20**	Somalia	Khalif Mumin Tohow	58	minister of justice of hirshabelle state (since 2017)	Somalia (Mogadishu)	https://www.aljazeera.com/news/2020/4/12/somali-state-minister-dies-from-coronavirus
**18-Apr-20**	Guinea	Sékou Kourouma	63	minitser government secretary general (since 2018)	Guinea (Conakry)	https://www.bloomberg.com/news/articles/2020-04-19/two-guinean-government-officials-die-after-getting-coronavirus
**03-May-20**	Niger	Mohamed Ben Omar	55	minister of labour	Niger (Niamey)	https://www.trtworld.com/africa/niger-labour-minister-dies-from-coronavirus-public-tv-36019
**25-May-20**	Somalia	Ismail Gamadiid	59	minister of agriculture, the environment, and climate change of puntland (since 2019)	Somalia (Mogadishu)	https://www.garoweonline.com/en/news/puntland/somalia-puntlands-minister-for-agriculture-dies-of-coronavirus-in-mogadishu
**08-Jun-20**	Burundi	Pierre Nkurunziza	55	president	Karuzi (Burundi)	https://www.theguardian.com/world/2020/jun/12/pierre-nkurunziza-obituary
**13-Jun-20**	Bangladesh	Mohammed Nasim	72	minister of Health and Family Affair	Bangladesh (Dhaka)	https://www.dhakatribune.com/bangladesh/politics/2020/06/13/mohammed-nasim-a-life-in-politics
**13-Jun-20**	Bangladesh	Sheikh Md Abdullah	74	minister of state of religious affairs	Bangladesh (Dhaka)	http://www.uniindia.com/b-desh-state-minister-for-religious-affairs-sheikh-md-abdullah-passes-away/world/news/2037783.html
**29-Jul-20**	Zimbabwe	Perrance Shiri	65	minister of lands, agriculture and rural resettlement of zimbabwe (since 2017)	Zimbabwe (Harare)	https://www.thesouthafrican.com/news/zimbabwe/who-is-perrance-shiri-black-jesus-dead-29-july-2020/
**29-Jul-20**	Bosnia and Herzegovina	Salko Bukvarević	53	minister for veterans and disabled veterans (since 2015)	Bosnia & Herzegovina (Sarajevo)	https://www.kosmo.at/schock-in-bosnien-herzegowina-minister-an-corona-gestorben/
**20-Sep-20**	Uzbekistan	Uktam Barnoev	56	deputy prime minister (since 2020)	Germany	https://www.rferl.org/a/uzbek-deputy-pm-barnoev-dies-from-covid-19-complications/30849707.html
**23-Sep-20**	India	Suresh Angadi	65	minister of state for railways of india (since 2019)	India (New Delhi)	https://www.timesnownews.com/india/article/i-am-doing-fine-taking-advice-of-doctors-suresh-angadi-s-last-tweet-after-he-tested-covid-19-positive/657061
**13-Dec-20**	Eswatini	Ambrose Mandvulo Dlamini	52	prime minister (since 2018)	South Africa	https://www.reuters.com/article/us-health-coronavirus-eswatini-idUSKBN28N0VJ
**19-Dec-20**	Uganda	Kirunda Kivejinja	85	minister of east african community affairs (since 2016)	Uganda (Kampala)	https://www.aa.com.tr/en/africa/uganda-s-2nd-deputy-premier-succumbs-to-covid-19/2083026
**12-Jan-21**	Malawi	Lingson Belekanyama	n/a	minister of local government and rural development (since 2019)	Malawi (Lilongwe)	https://www.monitor.co.ug/uganda/news/two-malawi-ministers-die-of-covid-19--3255066
**12-Jan-21**	Malawi	Sidik Mia	55	minister of transport and public works (since 2020 and 2010–2014)	Malawi	https://www.monitor.co.ug/uganda/news/two-malawi-ministers-die-of-covid-19--3255066
**15-Jan-21**	Zimbabwe	Ellen Gwaradzimba	60	Manicaland Provincial Affairs Minister	Zimbabwe (Harare)	https://www.zbcnews.co.zw/breaking-minister-gwaradzimba-dies/
**18-Jan-21**	Eswatini	Christian Ntshangase	51	minister of public service	Eswatini	https://www.zimlive.com/2021/01/24/eswatinis-labour-and-social-security-minister-dies-from-covid-19/
**20-Jan-21**	Zimbabwe	Sibusiso Moyo	60	minister of foreign affairs (since 2017)	Zimbabwe (Harare)	https://www.aa.com.tr/en/africa/zimbabwe-fills-cabinet-slots-created-by-covid-19/2138802
**21-Jan-21**	South Africa	Jackson Mthembu	62	minister in the presidency (since 2019)	South Africa (Johannesburg)	https://edition.cnn.com/2021/01/21/africa/south-africa-minister-covid-death-intl/index.html
**22-Jan-21**	Zimbabwe	Joel Matiza	60	minister of transport, communication and infrastructural development (since 2018)	Zimbabwe (Harare)	https://www.aa.com.tr/en/africa/zimbabwe-coronavirus-claims-transport-minister/2120095
**25-Jan-21**	Eswatini	Makhosi Vilakati	n/a	minister of labour	Eswatini	https://www.aa.com.tr/en/africa/eswatini-s-labor-minister-dies-of-covid-19/2121573
**26-Jan-21**	Colombia	Carlos Holmes Trujillo	69	minister of defense (since 2019)	Colombia (Bogotá)	https://www.nbcnews.com/news/latino/colombia-s-defense-minister-carlos-holmes-trujillo-dies-covid-age-n1255660

03-Feb-21	Senegal	Abdoul Aziz Mbaye	66	minister of culture (since 2012)	Senegal (Dakar)	https://afrique.latribune.fr/politique/2021-02-05/senegal-defenseur-engage-pour-une-afrique-resiliente-abdoul-aziz-mbaye-nous-a-quittes-a-l-age-de-67-ans-876997.html
13-Jul-20	Yemen	Hasan al-Lawzi	60	minister of information	Egypt (Cairo)	https://www.saba.ye/en/news3102675.htm

Tentative list of African Ministers and heads of state who had COVID-19 (excluding deaths)

Note: this list is unlikely to be comprehensive and should be treated with caution.

country	***name***	***position***	***source***
**South Africa**	Zweli Mkhize	health minister	https://www.usnews.com/news/world/articles/2020-10-18/south-africas-health-minister-tests-positive-for-covid-19 -
**South Africa**	Nosiviwe Mapisa-Nqakul	defense minister	https://www.latimes.com/world-nation/story/2020-10-02/trump-joins-growing-list-of-virus-infected-world-leaders -
**South Africa**	Gwede Mantashe	mineral resources and energy minister	https://www.latimes.com/world-nation/story/2020-10-02/trump-joins-growing-list-of-virus-infected-world-leaders -
**South Africa**	Thulas Nxesi	labor minister	https://www.latimes.com/world-nation/story/2020-10-02/trump-joins-growing-list-of-virus-infected-world-leaders -
**South Africa**	Ebrahim Patel	Trade, Industry, and Competition Minister	The Mercury - Factiva - Document MERCRY0020200727eg7r0000x - Minister tests positive for Covid-19
**Tunisia**	Othman Jerandi	foreign minister	https://www.aa.com.tr/en/africa/tunisias-foreign-minister-tests-positive-for-covid-19/2121608 -
**Tunisia**	Ali Kooli	minister of economy and finance	https://www.jeuneafrique.com/1120464/societe/un-an-de-covid-19-en-afrique-politiques-artistes-sportifs-ceux-qui-lont-eu-ceux-qui-lont-vaincu/ -
**Tunisia**	René Trabelsi	minister or tourism	https://www.jeuneafrique.com/1120464/societe/un-an-de-covid-19-en-afrique-politiques-artistes-sportifs-ceux-qui-lont-eu-ceux-qui-lont-vaincu/ -
**Morrocco**	Abdelkader Amara	Minister of Equipment	https://www.jeuneafrique.com/1120464/societe/un-an-de-covid-19-en-afrique-politiques-artistes-sportifs-ceux-qui-lont-eu-ceux-qui-lont-vaincu/ -
**Morrocco**	Aziz Rabbah	Minister of Energy	https://www.jeuneafrique.com/1120464/societe/un-an-de-covid-19-en-afrique-politiques-artistes-sportifs-ceux-qui-lont-eu-ceux-qui-lont-vaincu/ -
**Benin**	Romual Wadagni	Minister of the Economy	https://www.jeuneafrique.com/1120464/societe/un-an-de-covid-19-en-afrique-politiques-artistes-sportifs-ceux-qui-lont-eu-ceux-qui-lont-vaincu/ -
**Burkina Faso**	Alpha Barry	Minister of Foreign Affairs	https://www.jeuneafrique.com/1120464/societe/un-an-de-covid-19-en-afrique-politiques-artistes-sportifs-ceux-qui-lont-eu-ceux-qui-lont-vaincu/ -
**Burkina Faso**	Idani oumarou	Minister of Mines	https://www.jeuneafrique.com/1120464/societe/un-an-de-covid-19-en-afrique-politiques-artistes-sportifs-ceux-qui-lont-eu-ceux-qui-lont-vaincu/ -
**Burkina Faso**	Harouna Kaboré	Minister of Trade	https://www.jeuneafrique.com/1120464/societe/un-an-de-covid-19-en-afrique-politiques-artistes-sportifs-ceux-qui-lont-eu-ceux-qui-lont-vaincu/ -
**Burkina Faso**	Stanislas Ouaro	Minister of Education	https://www.jeuneafrique.com/1120464/societe/un-an-de-covid-19-en-afrique-politiques-artistes-sportifs-ceux-qui-lont-eu-ceux-qui-lont-vaincu/ -
**Burkina Faso**	Siméon Sawadogo	Minister of Environment	https://www.jeuneafrique.com/1120464/societe/un-an-de-covid-19-en-afrique-politiques-artistes-sportifs-ceux-qui-lont-eu-ceux-qui-lont-vaincu/ -
**Côte d'Ivoire**	Patrick Ani	President's chief of staff	https://www.jeuneafrique.com/1120464/societe/un-an-de-covid-19-en-afrique-politiques-artistes-sportifs-ceux-qui-lont-eu-ceux-qui-lont-vaincu/ -
**Côte d'Ivoire**	Hamed Bakayojo	Prime minister	https://www.jeuneafrique.com/1120464/societe/un-an-de-covid-19-en-afrique-politiques-artistes-sportifs-ceux-qui-lont-eu-ceux-qui-lont-vaincu/ -
**Guinea Bissau**	Umaro Sissoco Embalo	President	https://www.jeuneafrique.com/1120464/societe/un-an-de-covid-19-en-afrique-politiques-artistes-sportifs-ceux-qui-lont-eu-ceux-qui-lont-vaincu/ -
**Guinea Bissau**	Nuno Gomes Nabiam	Prime Minister	https://www.africanews.com/2020/04/30/guinea-bissau-pm-3-others-test-positive-for-covid-19/ -
**Guinea Bissau**	Antonio Deuna	Health Minister	https://www.africanews.com/2020/04/30/guinea-bissau-pm-3-others-test-positive-for-covid-19/ -
**Guinea Bissau**	Botche Candé	Interior Minister	https://www.africanews.com/2020/04/30/guinea-bissau-pm-3-others-test-positive-for-covid-19/ -
**Guinea Bissau**	Mario Fambe	Secretary of State for Public Order	https://www.africanews.com/2020/04/30/guinea-bissau-pm-3-others-test-positive-for-covid-19/ -

**Guinea Bissau**	Monica Boiro	Secretary of State for Regional Integration	https://www.africanews.com/2020/04/30/guinea-bissau-pm-3-others-test-positive-for-covid-19/ -
**Burundi**	Thaddée Ndikumana	Minister of Health	https://www.jeuneafrique.com/1120464/societe/un-an-de-covid-19-en-afrique-politiques-artistes-sportifs-ceux-qui-lont-eu-ceux-qui-lont-vaincu/ -
**CAR**	Henri-Marie Dondra	Minister of Finance	https://www.jeuneafrique.com/1120464/societe/un-an-de-covid-19-en-afrique-politiques-artistes-sportifs-ceux-qui-lont-eu-ceux-qui-lont-vaincu/ -
**DRC**	Acacia Bandubola	Minister of the Economy	https://www.jeuneafrique.com/1120464/societe/un-an-de-covid-19-en-afrique-politiques-artistes-sportifs-ceux-qui-lont-eu-ceux-qui-lont-vaincu/ -
**Botswana**	Mokgweetsi Masisi	President	https://www.jeuneafrique.com/1120464/societe/un-an-de-covid-19-en-afrique-politiques-artistes-sportifs-ceux-qui-lont-eu-ceux-qui-lont-vaincu/ -
**The Gambia**	Isatou Toura	Vice President	https://www.latimes.com/world-nation/story/2020-10-02/trump-joins-growing-list-of-virus-infected-world-leaders -
**The Gambia**	Fatou Kinteh	minister for women’s affairs, children and social welfar	Reuters - Factiva - Document LBA0000020200805eg8503t9p - UPDATE 1-Gambia imposes curfew as coronavirus cases surge 60% in a week
**The Gambia**	Mambury Njie	Finance Minister	RFI - Factiva - Document AFNWS00020200803eg830093z - Three Gambia Government Ministers Test Positive for Covid-19
**The Gambia**	Fafa Sanyang	Energy Minister	RFI - Factiva - Document AFNWS00020200803eg830093z - Three Gambia Government Ministers Test Positive for Covid-19
**The Gambia**	Amie Fabureh	Agriculture Minister	RFI - Factiva - Document AFNWS00020200803eg830093z - Three Gambia Government Ministers Test Positive for Covid-19
**Ghana**	Kwaku Agyeman Manu	Health Minister	https://www.africanews.com/2020/06/15/ghana-health-minister-tests-positive-for-covid-19// -
**Mauritania**	unnamed	minister	BBC - Factiva - Document BBCMEP0020200618eg6i0035x - Covid-19 Roundup: Middle East and North Africa 18 June 2020
**Nigeria**	Geoffrey Onyeama	foreign minister	BBC - Factiva - Document BBCAP00020200720eg7k000gp - BBCM Africa Watchlist for 20 July
**Nigeria**	Abba Kyari	Chief of Staff to the President of Nigeria (since 2015)	https://www.economist.com/middle-east-and-africa/2020/04/23/abba-kyari-who-tried-to-clean-up-nigeria-dies-of-covid-19 -
**South Sudan**	Riek Machar	vice president	http://apanews.net/en/news/sudan-10-cabinet-ministers-test-positive-for-covid-19 -
**South Sudan**	Angelina Teny	defence minister	http://apanews.net/en/news/sudan-10-cabinet-ministers-test-positive-for-covid-19 -
**South Sudan**	unnamed	minister 2	http://apanews.net/en/news/sudan-10-cabinet-ministers-test-positive-for-covid-19 -
**South Sudan**	unnamed	minister 3	http://apanews.net/en/news/sudan-10-cabinet-ministers-test-positive-for-covid-19 -
**South Sudan**	unnamed	minister 4	http://apanews.net/en/news/sudan-10-cabinet-ministers-test-positive-for-covid-19 -
**South Sudan**	unnamed	minister 5	http://apanews.net/en/news/sudan-10-cabinet-ministers-test-positive-for-covid-19 -
**South Sudan**	unnamed	minister 6	http://apanews.net/en/news/sudan-10-cabinet-ministers-test-positive-for-covid-19 -
**South Sudan**	unnamed	minister 7	http://apanews.net/en/news/sudan-10-cabinet-ministers-test-positive-for-covid-19 -
**South Sudan**	unnamed	minister 8	http://apanews.net/en/news/sudan-10-cabinet-ministers-test-positive-for-covid-19 -
**South Sudan**	unnamed	minister 9	http://apanews.net/en/news/sudan-10-cabinet-ministers-test-positive-for-covid-19 -
**Zambia**	Chitalu Chilufya	Health Minister	The East African - Zambia's Health minister tests positive for Covid-19
**Zambia**	Information Minister	Dora Siliya	The East African - Covid-19: Zambian minister tests positive
